# Eugene (Gene) Stern Paykel, MD, FRCPsych, FAcadMedSci

**DOI:** 10.1192/bjb.2024.89

**Published:** 2025-04

**Authors:** Peter Tyrer

Formerly Professor of Psychiatry, University of Cambridge, UK

**Figure:**
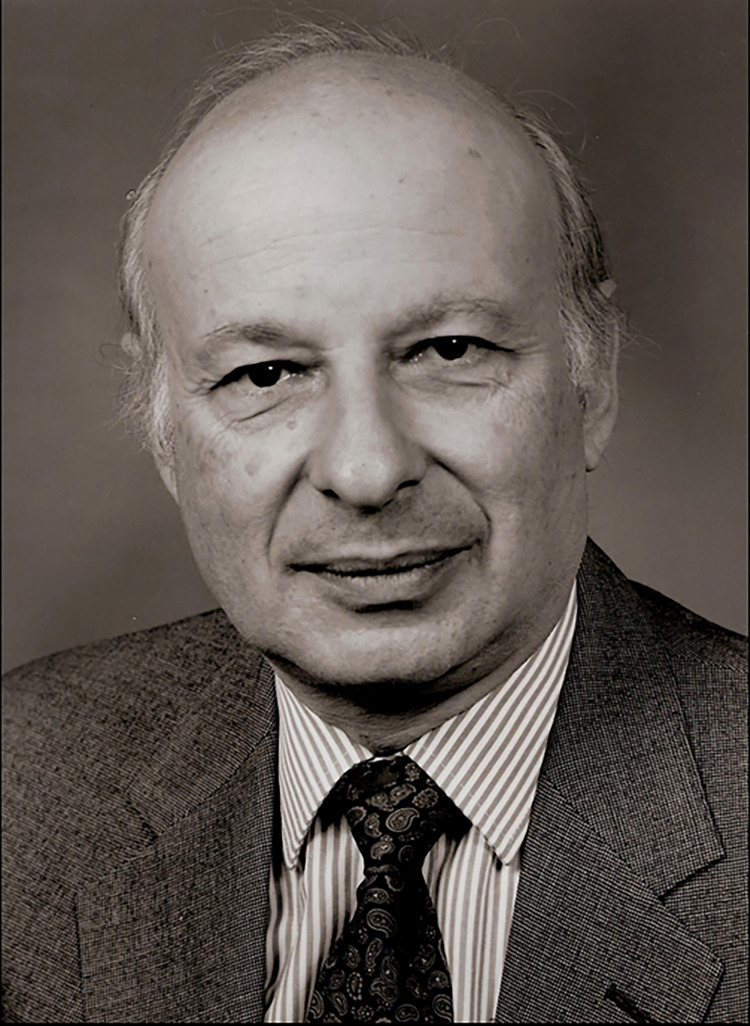

Gene Paykel, who died on 3 September 2023, aged 88, was a wise and distinguished psychiatrist who made many contributions to the fields of affective disorders and psychopharmacology. In retrospect, his views can be seen to be balanced, fair and well judged. His early research carried out in 1966 at Yale University on life events and classification of affective disorders showed excellent methodological rigour and is still widely cited.^1^ Puzzled by the high frequency of persistence and relapse in depression, his curiosity was stimulated by the many different settings in which depression arose, sometimes resolved, but at other times stayed far too long.

Working from St George's Hospital and Medical School in London, he carried out a number of controlled studies of the effectiveness of different types of antidepressant medication. He also led a controlled study of the effectiveness of community psychiatric nurses working with non-psychotic patients.

Later, he chaired the Defeat Depression Campaign in the UK between 1992 and 1996, which led to greater understanding of the subject among the general population, including the interesting addition that most patients thought antidepressant drugs were addictive, so foreshadowing more recent developments.

His balanced judgement and comprehensive knowledge made him a natural choice both to edit journals and to chair organisations. He was the founding editor (with George Winokur) of the *Journal of Affective Disorders* and later followed Michael Shepherd as editor of *Psychological Medicine* from 1994 to 2006. He was President of the British Association of Psychopharmacology at a critical early stage in its history and an important member of the International Marcé Society for Perinatal Mental Health in connection with his research on childbearing.

Gene was born on 9 September 1934 in Auckland, New Zealand, to Joshua (Joe), a businessman, and Eva, a concert pianist, both of whom came from Jewish families that had migrated to New Zealand from Russia. The family name of Paykel is very well-known in New Zealand as a supplier of kitchen appliances, and although his path diverged greatly from his commercial relatives, both had solid and long-lasting useful foundations. He was the eldest of three children, having two younger sisters.

He trained at the University of Otago Medical School in Dunedin and, after house jobs in New Zealand, migrated to the UK, travelling through the Panama Canal on a cargo ship on which he was the ship's doctor. After further house jobs and success in postgraduate examinations in Scotland and England, he opted to train in psychiatry and, like many others at that time, was attracted to the Maudsley Hospital in London. At the Maudsley, under the sceptical gaze of Aubrey Lewis and Michael Shepherd, he quickly learned the disciplines of careful reporting, independent verification and editorial judgement. In 1966, he moved to the USA to a research post at Yale University Medical School. There he was supervised by and soon became involved in joint work with Gerald Klerman. Together they co-founded the Depression Research Unit at Yale, where they were joined by Myrna Weissman and other distinguished American collaborators.

He married Margaret (Maggie) Melrose, a librarian, in 1969 and they had two sons. After his research experience in the USA, he and his wife returned to the UK in 1971 and he took up an academic appointment at St George's Hospital, London, where he became Professor of Psychiatry. In 1985 he was appointed Head of Psychiatry at the University of Cambridge, in succession to Sir Martin Roth. He managed to turn the Department of Psychiatry into a successful, highly rated academic powerhouse. He was elected a Fellow of Gonville & Caius College, Cambridge in 1986 and a Fellow of the Academy of Medical Sciences in 1998. At Cambridge, he continued his research on life events and affective disorders, extending this to the puerperium, and this covered many disciplines.

I knew Gene for over 50 years and throughout this time he was always impeccably polite, sensitive and generous in his advice (qualities that made him an excellent editorial colleague), and supportive and encouraging to others, especially younger researchers. At Gonville & Caius College he was well liked and loved being part of the Cambridge establishment, although, as a New Zealander, was always able to maintain a level of amused independence. He also had a most infectious laugh that doubled me up. He had no need for incessant drive or ambition to be highly praised; what he did he did so conscientiously and well. We often say that someone we know well is worthy, then have to follow it by asking ‘worthy of what?’. Gene Paykel was the embodiment of unqualified worthiness, a true champion. Gene is survived by Maggie, his devoted no-nonsense wife, who supported his work for many years, and his two sons, Jon and Nick.
